# Validity of a virtual reality endoscopic retrograde cholangiopancreatography simulator: can it distinguish experts from novices?

**DOI:** 10.3389/fsurg.2023.1289197

**Published:** 2023-12-06

**Authors:** Konstantinos Georgiou, Nikola Boyanov, Pantelis Antonakis, Dimitrios Thanasas, Gabriel Sandblom, Lars Enochsson

**Affiliations:** ^1^ 1st Department of Propaedeutic Surgery, Hippokration General Hospital of Athens, Athens Medical School, National and Kapodistrian University of Athens, Athens, Greece; ^2^ Medical Simulation Training Center, Research Institute of Medical University of Plovdiv, Plovdiv, Bulgaria; ^3^ 2nd Department of Surgery, Medical School, Aretaieion Hospital, National and Kapodistrian University, Athens, Greece; ^4^ Medical Physics Laboratory Simulation Center, Athens Medical School, National and Kapodistrian University of Athens, Athens, Greece; ^5^ Department of Clinical Science and Education Södersjukhuset, Department of Surgery, Södersjukhuset, Karolinska Institutet, Stockholm, Stockholm, Sweden; ^6^ Department of Surgical and Perioperative Sciences, Surgery, Umeå University, Umeå, Sweden

**Keywords:** endoscopic retrograde cholangiopancreatography (ERCP), simulation, endoscopy, performance, experts, novices

## Abstract

**Background:**

There is a lack of evidence regarding the effectiveness of virtual simulators as a means to acquire hands-on exposure to endoscopic retrograde cholangiopancreatography (ERCP). The present study aimed to assess the outcome and construct validity of virtual ERCP when training on the GI II Mentor simulator.

**Methods:**

A group of seven experienced endoscopists were compared with 31 novices. After a short introduction, they were requested to carry out three virtual ERCP procedures: diagnosing and removing a common bile duct (CBD) stone; diagnosing and taking brush cytology from a hilar stenosis; and, finally, diagnosing and treating a cystic leakage with a BD stent. For each task, the total time required to complete the task, time required to correctly view the papilla, total time of irradiation, time to deep cannulation, time to define diagnosis, time to complete sphincterotomy, and time to complete the respective intervention were measured. Cannulation of the BD, correct diagnosis, sphincterotomy, and time to complete intervention were assessed by an assessor blinded to the status of the endoscopist who performed the virtual ERCP.

**Results:**

The time required to visualize the papilla and to cannulate deeply when removing the BD stone was significantly shorter for the experts (both *p* < 0.05). The time to visualize the papilla, cannulate deeply, reach a diagnosis, complete sphincterotomy, and complete the intervention was significantly shorter for the experts when managing cystic leakage (all *p* < 0.05). In diagnosing and taking brush cytology from a hilar stenosis, there was only a trend toward the experts needing less time for the deep cannulation of the BD (*p* = 0.077).

**Conclusion:**

The performance differed between experts and novices, especially in the management of cystic leakage. This corroborates the construct validity of the GI II Mentor simulator.

## 1. Introduction

Endoscopic retrograde cholangiopancreatography (ERCP) is a technically demanding procedure with a high risk for serious adverse events. Visceral perforation and bleeding are sometimes seen after ERCP, but the most feared complication is that of post-ERCP pancreatitis (PEP), with a rate of 3.5%–5% (1). It has been suggested that higher endoscopist case volumes are associated with safer ERCP and successful outcomes, and therefore, there is an urgent need to provide opportunities for hands-on training for all endoscopists and surgeons performing ERCP (2, 3).

Because of its technical complexity and risk, training ERCP in simulation-based settings is warranted and has the potential to reduce the hazards related to the early learning curve than most other standard procedures (4). Training in more advanced therapeutic procedures such as ERCP is usually not provided by mechanical simulators and therefore virtual reality (VR) simulators are crucial. Ekkelenkamp et al. (5), in a systematic review, concluded that simulator training is complementary to patient-based learning and is useful in the early training phase in enhancing the early learning curve and avoiding patient hazards. However, most studies regarding the beneficial effects of VR simulation endoscopic training have almost exclusively been performed in virtual gastroscopy and colonoscopy.

Different approaches have been suggested to be used for ERCP simulators, but regardless of whether it is a mechanical simulator, an *in vivo* or *ex vivo* model, or a virtual simulator, all studies but one (6) failed to fulfill the criteria of a robust validation study as suggested by Downing and Haladyna (7). However, it is important to stress that the simulator constructed by Jirapinyo et al. (6), although thoroughly validated, is a mechanical simulator that has its main role in the preclinical setting as these focus on the technical aspects of basic ERCP skills only. More specifically, in the case of VR ERCP simulation, there is only one small underpowered study that showed the construct and face validity of a high-fidelity ERCP simulator (GI Mentor II, Surgical Science Sweden AB, Gothenburg, Sweden) (4).

**Figure 1:**
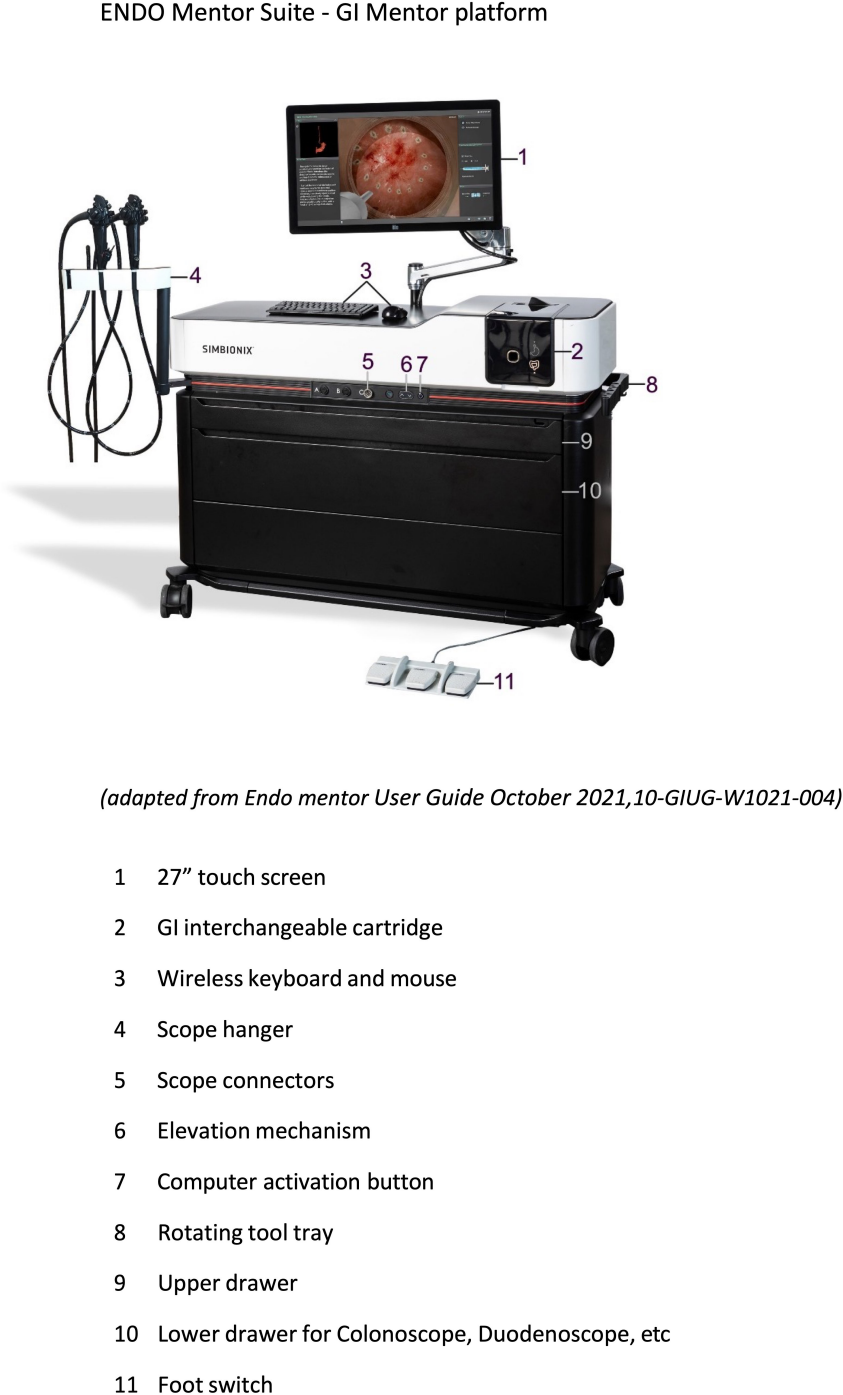
ENDO Mentor Suite—GI Mentor platform. Figures are publicly available from the Surgical Science Website.

The primary outcome of this study was to assess the construct validity of three different VR ERCP procedures of increasing difficulty of the GI Mentor II (Figure 1) by assessing seven clinically relevant parameters and to compare the performance of a group of novices to those from experts.

## 2. Materials and methods

### 2.1. Participants and simulation procedures

The expert group consisted of seven experts in ERCP (with experience of more than 1,000 ERCP procedures each so far). Before participating in this study, they all completed an informed consent form. To begin with, they were briefed on the ERCP modules in GI Mentor II and on its add-ons (i.e., guidewires). Following that, they performed a bile duct (BD) cannulation case three times to get acquainted with the simulator. Then, the experts completed the following three virtual ERCP procedures of increasing difficulty:

a. ERCP procedure 1: BD stone removal (ERCP Module 1, Case study 4)

**Figure 2:**
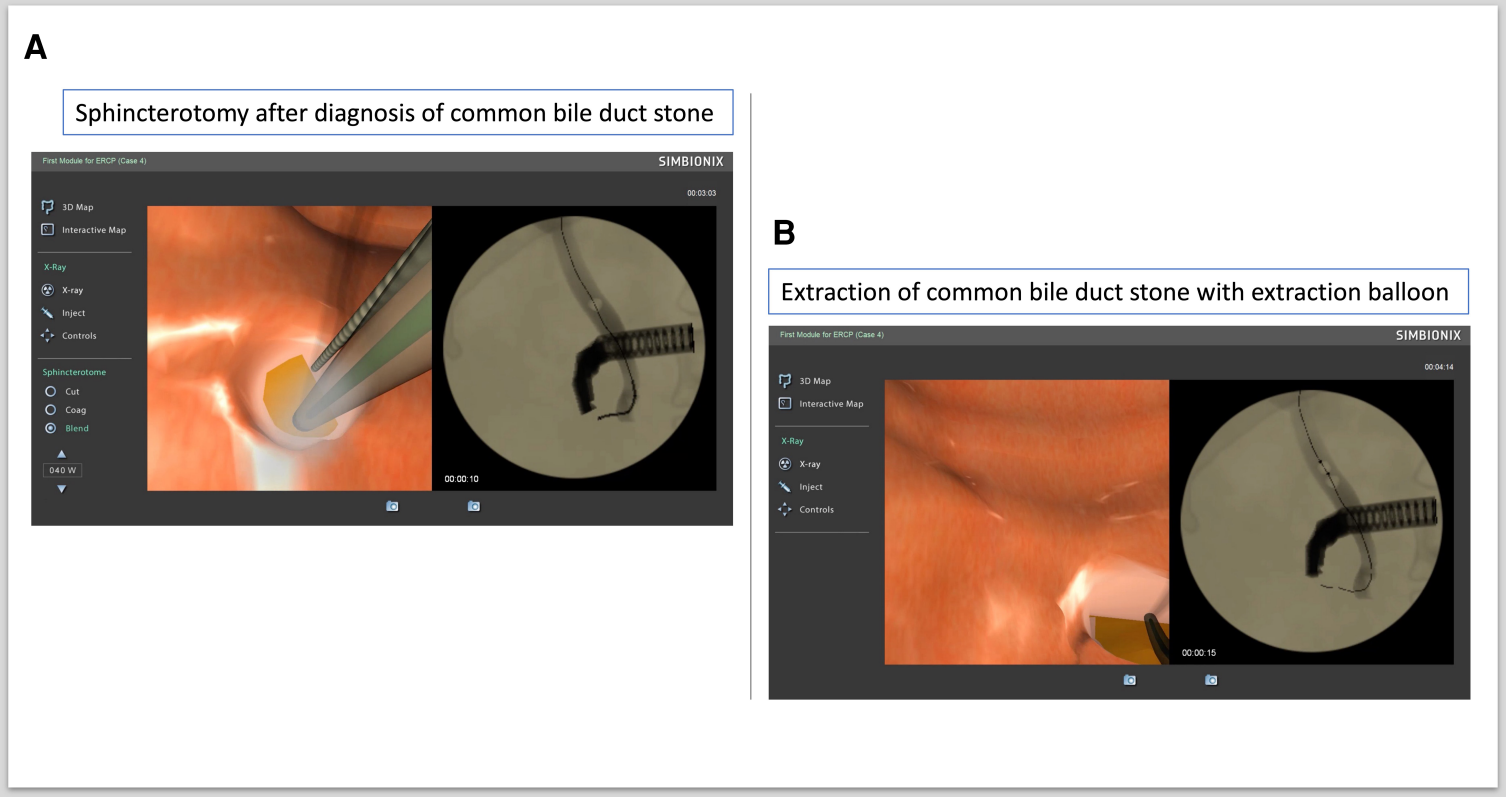
Extraction of common bile duct (CBD) stone. (**A**) Sphincterotomy after diagnosis of CBD stone. (**B**) Extraction of CBD stone with extraction balloon.

In this procedure, the BD was cannulated with a sphincterotomy catheter. Then, a guidewire was inserted into the common bile duct (CBD) and contrast injected to get the proper diagnosis of a BD stone. A sphincterotomy was then performed (Figure 2A). The sphincterotome was then removed and replaced with an extraction balloon to remove the CBD stone (Figure 2B).

b. ERCP procedure 2: hilar stenosis and performing brush cytology (ERCP Module 1, Case study 2)

**Figure 3:**
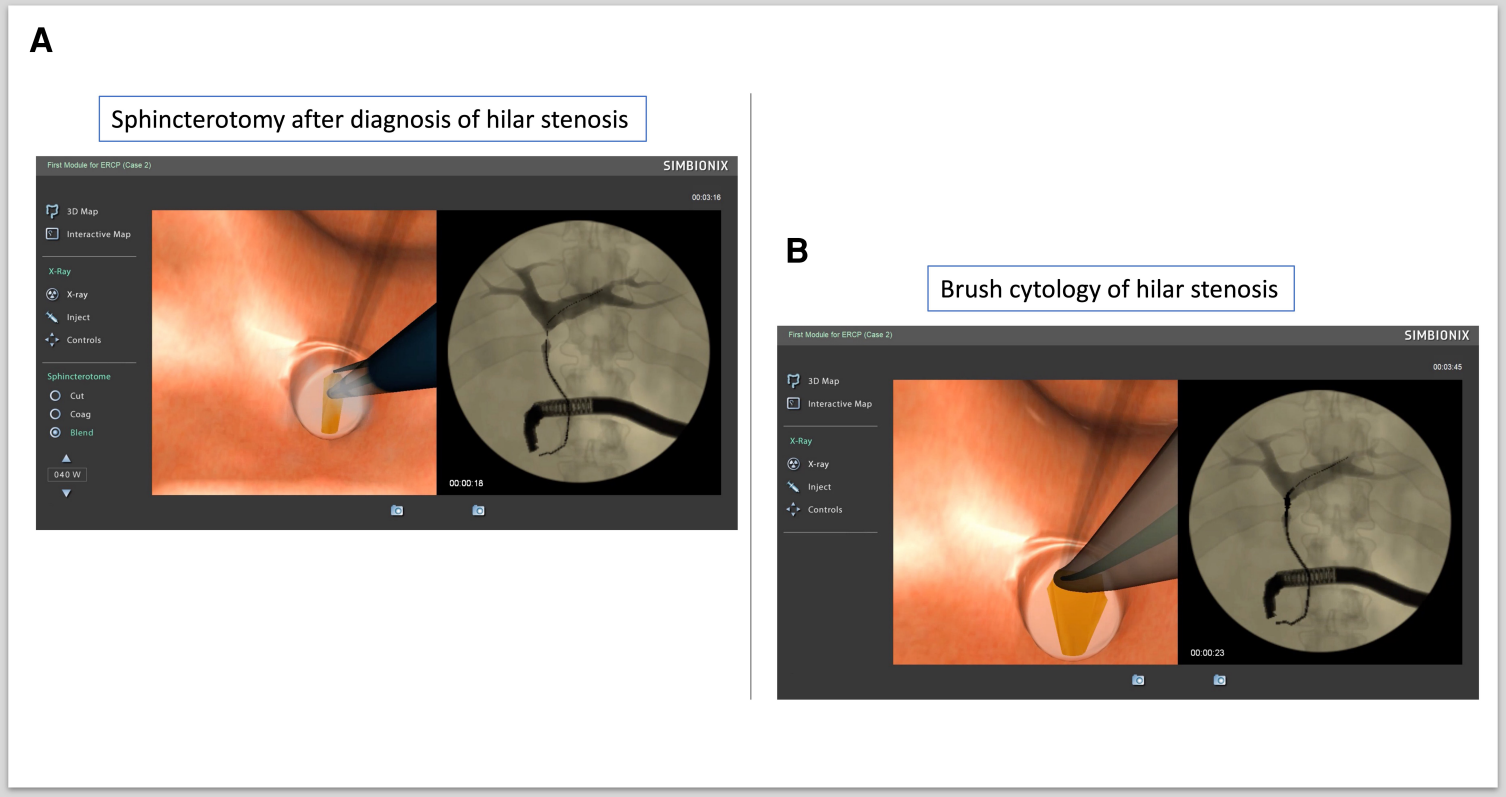
Brush cytology of hilar stenosis. (**A**) Sphincterotomy after diagnosis of hilar stenosis. (**B**) Brush cytology of hilar stenosis.

The CBD was cannulated, a guidewire was inserted and contrast injected, and hilar stenosis was diagnosed. Then, a sphincterotomy was performed (Figure 3A). The sphincterotome was removed and replaced with a cytology brush with which brush cytology samples were collected from the hilar stenosis (Figure 3B).

c. ERCP procedure 3: diagnosis of cystic leakage and treatment with BD stent placement (ERCP Module 2, Case study 4)

**Figure 4:**
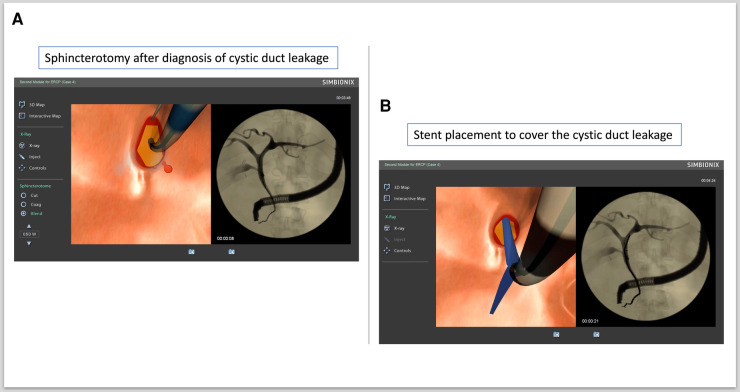
Diagnosing and treating cystic duct leakage. (**A**) Sphincterotomy after diagnosis of cystic duct leakage. (**B**) Stent placement to cover cystic duct leakage.

Cannulation of the CBD and sphincterotomy after the diagnosis of cystic duct leakage were performed (Figure 4A). Plastic stent placement was carried out to cover the cystic duct leakage (Figure 4B).

For each of the abovementioned tasks, an automatic report was stored on the simulator and the following parameters were extracted for analysis:

1. Total time of the procedure (s)
2. Time to view the papilla correctly (s)
3. Total time of irradiation (s)

In addition, all task sessions were videotaped, and one expert coauthor (LE), blinded to the name and role of the respective endoscopist, reviewed the videos and manually extracted them:

4 Time to deep cannulation of the BD (s)
5 Time to diagnosis (s)
6 Time to sphincterotomy (s)
7 Time to complete intervention (s)

The novice group initially consisted of 35 novice endoscopists (gastroenterologists or residents in gastroenterology). Four of them discontinued the trials for different reasons, so finally 31 novices were included in the analysis. They also gave informed consent and completed a questionnaire regarding background information and computer gaming experience. As for the expert group, they followed an introductory lecture regarding the basics of ERCP procedures and a hands-on acquaintance with the simulator. They also performed on the simulator the same three procedures consequently and in the same order as those of the novice group.

The same seven parameters were identically calculated, and both groups were unaware of the assessment metrics. The study is registered with the Research Registry (researchregistry.com) under the unique identification number (UIN) researchregistry8906. All participants participated voluntarily.

### 2.2. Statistical analysis

JMP Pro 16.0.0 statistical software (SAS Institute Inc, Cary, NC, USA) was employed for the statistical analysis. Continuous data conforming to a normal distribution are presented as mean ± SD and were analyzed using the Student's *t*-test. Pearson's chi-squared test was used for the comparison of categorical variables. All results were considered statistically significant at *p* < 0.05. The statistical analyses were performed by one of the coauthors (LE).

## 3. Results

The demographics and characteristics of the two groups are presented in Table 1.

**Table 1:** Demographics and characteristics of the two groups.

		Experts (*N*)	Students (*N*)	*p*
Sex	Females	0	16	**0**.**0125**
	Males	7	15	
Age (years)	40+	3	9	0.3653
	30–40	4	15	
	20–30	0	7	
Already a specialist in gastroenterology	Yes	7	24	0.1639
	No	0	7	
Number of gastroscopies carried out	500+	7	20	0.1741
	200–500	0	8	
	100–200	0	0	
	Up to 100	0	3	
Number of colonoscopies carried out	500+	7	14	0.0736
	200–500	0	5	
	100–200	0	4	
	Up to 100	0	8	
Number of polypectomies carried out	100+	6	11	**0**.**0365**
	50–100	1	5	
	Up to 50	0	15	
Experience from video games	Yes	6	15	*0*.*0728*
	No	1	16	

Bold values indicate significant differences, i.e., the *p*-values are <0.05.

The outcome of the three ERCP procedures and the comparison between the two groups are presented in Tables 2–4.

**Table 2:** Parameters measured in bile duct (BD) stone removal (ERCP Module 1, Case study 4).

Parameters	Experts		Novices		*p*
	Mean	SD	Mean	SD	
1. Total time (s)	273	38	273	83	0.999
2. Time to view papilla correctly (s)	89	29	124	45	**0**.**023**
3. Total time of x-ray (s)	27	11	31	16	0.471
4. Time to deep cannulation (s)	127	27	160	54	**0**.**028**
5. Time to diagnosis (s)	139	29	167	54	0.068
6. Time to sphincterotomy (s)	173	31	192	60	0.249
7. Time to complete intervention (s)	238	35	246	80	0.661

Bold values indicate significant differences, i.e., the *p*-values are <0.05.

**Table 3:** Parameters measured in hilar stenosis (ERCP Module 1, Case study 2).

Parameters	Experts		Novices		*P*
	Mean	SD	Mean	SD	
1. Total time (s)	214	44	221	53	0.729
2. Time to view papilla correctly (s)	99	38	121	35	0.181
3. Total time of x-ray (s)	22	6	23	12	0.960
4. Time to deep cannulation (s)	116	41	151	41	0.077
5. Time to diagnosis (s)	129	42	157	42	0.141
6. Time to sphincterotomy (s)	148	45	171	45	0.240
7. Time to complete intervention (s)	180	44	198	50	0.370

**Table 4:** Parameters measured in cystic leakage (ERCP Module 2, Case study 4).

Parameters	Experts		Novices		*p*
	Mean	SD	Mean	SD	
1. Total time (s)	304	155	252	64	0.415
2. Time to view papilla correctly (s)	80	31	124	35	**0**.**008**
3. Total time of x-ray (s)	46	53	28	16	0.410
4. Time to deep cannulation (s)	77	33	149	42	**<0**.**001**
5. Time to diagnosis (s)	89	38	170	49	**0**.**001**
6. Time to sphincterotomy (s)	106	39	186	53	**0**.**001**
7. Time to complete intervention (s)	154	60	226	57	**0**.**018**

Bold values indicate significant differences, i.e., the *p*-values are <0.05.

As can be seen from Table 2 (BD stone removal), a significant difference between novices and experts was seen for “Time to view the papilla correctly” and “Time to deep cannulation” (*p* = 0.023 and *p* = 0.028, respectively). In the hilar stenosis procedure (Table 3), there were no statistical differences, although the “Time to deep cannulation” was close to reaching significance (*p* = 0.077). Finally, the statistical differences between experts and novices were most pronounced in the cystic leakage procedure (Table 4), as five out of the seven parameters measured showed statistically significant differences.

## 4. Discussion

ERCP is a technically demanding procedure for beginner endoscopists. Over the years, various ERCP-specific indicators and quantified criteria have been developed to help mentors assess the skills of novice endoscopists. Several endoscopic organizations have published quality measurement guidelines and recommendations, although their applicability in daily practice is limited (8).

In the present study, both groups completed the same three ERCP cases. Seven parameters (three automatically generated by the simulator and four manually derived after video inspection) were assessed to examine differences between experts and novices. The differences seen between experts and novices indicate that the model has adequate validity and that the experience acquired by the experts is reflected in a shorter time to accomplish some of the crucial steps, such as cannulation of the BD, in each procedure. We hypothesize that one of the main reasons why ERCP can be experienced by some as technically more difficult than both gastroscopy and colonoscopy is the visuospatial challenge due to the side-directed optics. We feel this is supported by the fact that time to view papilla correctly (s) takes 39% longer for novices compared to experts (Table 2), 22% (Table 3), and 55% (Table 4). The corresponding figures for time to deep cannulation (s) are 26% (Table 2), 30% (Table 3), and 93% (Table 4).

### 4.1. ERCP Module 1, Case 4 (BD stone)

The experts needed less time to view the papilla correctly and to cannulate the BD. There was also a trend that the experts arrived quicker at diagnosis. Cannulating the BD is generally considered a crucial step in ERCP (9). Although the simulated model does not assess all aspects of a successful procedure, it may be assumed that prolonged time to cannulate is associated with an increased risk of failed cannulation and PEP (10).

### 4.2. ERCP Module 1, Case 2 (hilar stenosis)

There was a non-significant trend in favor of the experts regarding time to cannulate the BD. However, regarding the other parameters, there were no differences. As this procedure in the simulator requires fewer skills with fewer technical manipulations than the other procedures, the relative advantage of acquired experience is reduced. Nevertheless, as in the BD stone procedure, the shorter time needed for the experts to cannulate the BD corroborates the criterion validity.

### 4.3. ERCP Module 2, Case 4 (cystic duct leakage)

This procedure is perhaps the most complex of the procedures for two reasons:

A. The participants have to inject more contrast to visualize the cystic duct bile leakage. That in itself is perhaps not that difficult, but it requires the experience and knowledge of how to detect a cystic duct leakage. This may not require advanced understanding but some experience in managing the situation.
B. It is a more complex procedure since it requires placing a BD stent correctly.

In addition, in this procedure, significant differences were observed almost everywhere between experts and novices, a finding that indicates construct validity.

Bitner et al. (4) in a small heterogenous sample [three postgraduate year (PGY) 1, three PGY gastroenterology fellows, three gastroenterologists, and three gastrointestinal (GI) surgeons] performed two procedures on a GI Mentor II simulator, namely, case A, which required stent placement with sphincterotomy for cystic duct leak after laparoscopic cholecystectomy. Second, participants performed case B, involving a pancreatic head mass, which necessitated CBD brushing and balloon dilation for stricture plus sphincterotomy and stent placement for duct decompression. These cases met the criteria for standard and advanced degrees of difﬁculty, respectively (11). They concluded that GI Mentor II demonstrated construct validity for ERCP based on select metrics as mean procedure time-defined skill levels. When the outcomes of the two cases were combined, beginners and experts differed based on the time to complete the procedure, reach the papilla, and use of fluoroscopy. Other ERCP-specific metrics failed to demonstrate construct validity, likely due to the small sample size. They suggested that prospective, multicenter trials will be required to demonstrate the predictive validity of the GI Mentor II for ERCP. In contrast to Bitner et al., in our study, we used both simulator-generated parameters (#1–3) and expert's judge-based custom metrics (#4–7) since they accurately present the exact moments of the procedure steps (time to BD cannulation, diagnosis, sphincterotomy). Furthermore, we used a bigger sample, but we seconded their suggestion that more studies are needed in the field.

Leung et al. (12) compared an ERCP mechanical simulator (EMS) to a VR one (GI Mentor II, ECS). Their sample size consisted of 18 trainees and 16 trainers who expressed their impressions using a questionnaire. Both trainers and trainees showed significantly greater increases in scores for EMS vs. ECS in facilitating the ERCP procedure, enhancing confidence in clinical ERCP. EMS scored significantly higher in realism and usefulness. However, the questionnaire evaluation is subjective and may provide answers affected by the varying experiences of the responders, even among the trainers. In our study, all the participants volunteered for study inclusion, which makes the data vulnerable to volunteer bias (13). The participants volunteering could be more motivated, which could obscure the difference in performance between novices and experts. Large sample sizes are required to show significant differences in patient clinical outcomes, which is rarely possible in a medical education study (14).

The differences seen between the experts and novices could be explained by factors other than the experience from *in vivo* ERCP, e.g., familiarity with the simulated model and a better ability to understand the construction of the simulated environment. Nevertheless, the difference observed between the groups indicates that the simulated model reflects a range of experiences that, at least in theory, could be overcome with simulated training. The outcome measures reflecting the greatest difference between the novices and experts, i.e., the time required to complete the procedures, are surrogate measures of the skills of the endoscopist.

### 4.4. Study limitations

ERCP is a technically demanding procedure and is nearly always carried out by advanced endoscopists with experience. Obviously, there will be differences in terms of the concept of a simulator and clinical practice. For instance, some cases on the simulator might appear to be more straightforward than in a real-life setting, which are considered to be more complex procedures (i.e., hilar stenosis). Therefore, to overcome such issues, more complex cases on the virtual simulator could be more effective for the trainee and check if the observed differences between the two groups are persisting.

Furthermore, a plethora of quality indices and measures have been proposed for ERCP (8) while our study focused only on some parameters of the intraprocedural procedure. Although the differences between experienced doctors and trainees in the simulated ERCP procedures are significant, they are rather small. We believe that these small differences do not fully reflect the differences in ERCP on real patients since the simulator, although excellent in many of the technical moments, has limitations in others such as the cannulation of the BD, which we experience is much more difficult in real ERCP. However, we feel that the great advantage that GI Mentor II has in ERCP simulation is that it forces the novice to really try to position him/herself correctly with the duodenoscope in relation to the papilla to succeed with the cannulation.

In addition, an increased number of participants should allow for a more precise categorization into novice, advanced beginners, proficient, and expert groups to examine the validity of this simulator to distinguish among them.

Therefore, although the present study confirmed the criterion validity of the simulator, more studies are surely needed to explore more in-depth whether simulation training affects the learning curve. Ideally, also, a measure of the tissue trauma and maneuvers potentially causing PEP should be provided. Quantifying such features is, however, very complicated.

## 5. Conclusion

Our study showed that the outcome from the GI Mentor II accurately discriminates between experts and novices. This supports the criterion validity, but more studies are needed to assess whether the simulator improves the outcome for endoscopists under training. Other preprocedure, intraprocedural, and postprocedure measures of performance than time to complete procedures should also be considered.

## Data Availability

The original contributions presented in the study are included in the article/Supplementary Material, further inquiries can be directed to the corresponding author.
